# Structure of TRAF Family: Current Understanding of Receptor Recognition

**DOI:** 10.3389/fimmu.2018.01999

**Published:** 2018-08-30

**Authors:** Hyun H. Park

**Affiliations:** College of Pharmacy, Chung-Ang University, Seoul, South Korea

**Keywords:** inflammation, innate immunity, TRAF family, structure, TRAF domain, protein interaction

## Abstract

Tumor necrosis factor receptor–associated factor (TRAF) proteins are key signaling molecules that function in various cellular signaling events including immune response, cell death and survival, development, and thrombosis. Their roles in cellular signaling are mediated mostly by direct interactions with various receptors via the TRAF domain. To determine how specific TRAF domains can interact with various receptors with a limited binding interface and how similar binding interfaces of TRAF family members can recognize their specific binding partners, extensive structural studies on TRAF family proteins have been conducted for several decades. In this review, we discuss the current understanding of the structural and molecular diversity of the TRAF domain and TRAF-binding motifs in many receptors according to available structural information.

## Introduction

**Figure 1:**
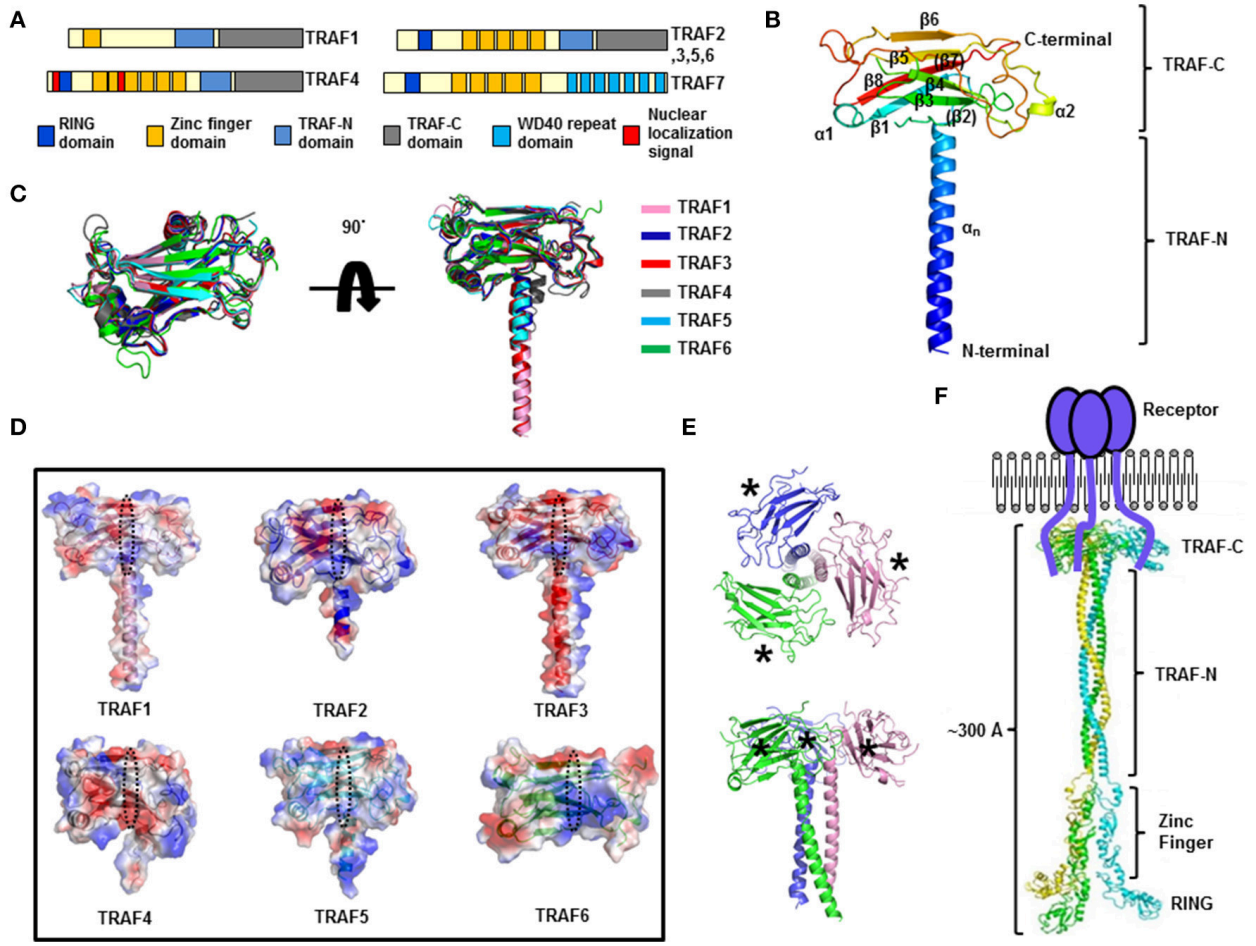
Structure of the TRAF family. **(A)** The domain boundary in TRAF family members. **(B)** A cartoon of the monomeric TRAF domain. The representative TRAF domain of TRAF1 is used to show the overall structure of this domain. The chain from the N to C terminus is colored blue to red. Secondary structures including helices and sheets are labeled. **(C)** Superposition of the structures of the TRAF domain. **(D)** Electrostatic surface representation of the TRAF domain in the TRAF family. PyMol (https://www.pymol.org/) was used to compute qualitative surface electrostatic potential. The receptor-binding region in a TRAF family member is indicated by the black dotted circle. **(E)** A cartoon of the trimeric TRAF domain. Different chains are shown separately in different colors. The top view (upper panel) and side view (lower panel) are presented. The asterisks indicate the receptor-binding region. **(F)** A model of the full-length structure of TRAF family members.

Tumor necrosis factor (TNF) receptor–associated factor (TRAF) proteins, which include seven family members (from TRAF1 to TRAF7) in mammals, are key signaling molecules that can transduce signals in various types of receptor-mediated cellular signaling, including tumor necrosis factor receptor (TNF-R), interleukin 1 receptor/Toll-like receptor (TLR), nucleotide-binding oligomerization domain-like receptor (NLR), RIG-I like receptor (RLR), and even cytokine receptor family signaling pathways, and play critical roles in the regulation of the immune system and apoptosis (Supplementary Table 1) (1–4). The main feature of TRAF family proteins (except for TRAF1) is the homology RING domain at the N terminus; this domain is found in many E3 ubiquitin ligases and constitutes the core of the ubiquitin ligase catalytic domain and is important for ligase activity (5, 6). Another feature of the members of the TRAF family (except for TRAF7) is the presence of a protein–protein interaction domain of ~230 amino acid residues, known as the TRAF domain, at the C terminus (Figure 1A). The TRAF domain is subdivided into two distinct subdomains: the TRAF-N domain, which is a coiled-coil domain, and the TRAF-C domain, which is composed of seven to eight anti-parallel β-strand folds. TRAF family members form a mushroom-like trimeric structure in solution via the TRAF domain, which is the functional unit of a TRAF (7, 8).

Depending on composition of this domain, TRAF proteins have two main functions: the E3 ubiquitin ligase function and scaffolding function. The scaffolding function of TRAF family members is based mainly on the TRAF domain, which can mediate interactions of various membrane receptors with diverse downstream effector molecules, primarily protein kinases, including IRAKs, RIP1, RIP2, TAK1, MEKK1, and ASK1 (9–12) and several ubiquitin ligases such as members of the cIAP family (13). Although a TRAF is a positive mediator of signaling events, the antagonistic roles of TRAFs in TNF-R and TLR signaling have been reported (8, 14). The E3 ligase activity of TRAFs has also been intensively studied, and substrates of each family have been identified (Supplementary Table 2) (6, 15–18). According to their roles in many critical signaling pathways, TRAFs are related to many human diseases, including cancer, autoimmunity, and inflammatory diseases, and have been suggested as suitable targets for therapeutic intervention (19–23). Because of their important biological roles, structural studies on the TRAF family have increased in number. Specifically, studies have examined how specific TRAF proteins can interact with various receptors through the limited binding interface and how the similar binding interfaces of each TRAF family member can recognize their binding partners. The structures of the TRAF domain of TRAF2, TRAF3, and TRAF6 and their receptor complexes were elucidated around year 2000 (24–26), and those of TRAF1, TRAF4, and TRAF5 and their receptor complexes were examined recently (27–32). In this review, we discuss the current understanding of the structural and molecular diversity of the TRAF domain according to available structural information.

## Structure of TRAF family

The presence of the TRAF domain, a ~180 amino acid protein-interacting domain, is a distinct feature of TRAF family proteins and six TRAF proteins (TRAF1–TRAF6) among the seven in the family, in accordance with this criterion, have been identified as the TRAF family in mammals (3). The TRAF domain can be subdivided into two distinct regions: the TRAF-N domain and TRAF-C domain. Various receptors bind to the TRAF-C domain, while various intracellular signaling molecules bind to the TRAF-N domain. Despite the structural similarity of TRAF domains, each TRAF protein has specific biological functions with specificity to the interacting partners: upstream receptors and downstream effector molecules. The structure of the TRAF domain of TRAF2 was first reported by Dr. Wu's group around 1999 (24), and the structure of TRAF6's TRAF domain was reported 3 years later by the same group (25). Since then, the structures of the TRAF domain of TRAF3 (27), TRAF5 (27), TRAF4 (28–30), and TRAF1 (31) have been reported. The TRAF structures revealed that the TRAF-N domain is a coiled-coil structure, and TRAF-C is composed of seven to eight anti-parallel β-sheet folds (Figure 1B). Structural alignment of all six TRAF family members shows that the TRAF-C domain is well-aligned, while the location and length of TRAF-N varies among TRAF family members (Figure 1C). Sequence analysis indicates that the length of TRAF-N varies in the family, whereas that of the TRAF-C domain is conserved: the length of TRAF-N of TRAF4 and TRAF6 is relatively shorter, while TRAF3 and TRAF5 are relatively longer (Supplementary Figure 1).

Although the overall structures are nearly identical, obvious structural differences have been observed. For example, the length and position of some loops in the TRAF domain of TRAF4 and TRAF6 differ from those of other TRAF family members (Figure 1C). Particularly, two loops connecting β5-β6 and β6-β7 of the TRAF4 TRAF domain are relatively longer than those of other TRAFs. The location of the TRAF-N coiled-coil domain also differs in that it is in the outer layer only in the structure of TRAF4 (Figure 1C). These slight differences in structure among the TRAF family members may be responsible for their functional differences. Characteristics of the electrostatic surface of the TRAF domain vary within the family, although the TRAF domains of TRAF1, TRAF2, TRAF3, and TRAF5 have similar overall features, with mixed positive and negative charges and several uncharged regions (Figure 1D). TRAF4 contains a more negatively charged surface in the middle of the receptor-binding region, whereas TRAF6 contains a more positively charged surface in the receptor-binding region (Figure 1D). Because the surface features often determine their mode of interactions with partners, the similar electrostatic surface of the TRAF domain among TRAF1, TRAF2, TRAF3, and TRAF5, namely, diversely charged surfaces, has been shown to be important for accommodating diverse receptors in the same binding pocket with similar modes of interaction. In contrast, different features on the binding surface of functionally different TRAFs, TRAF4, and TRAF6, indicate that TRAF4 and TRAF6 can accommodate different receptors with different modes of interactions.

In solution, the TRAF domain forms a stable functionally important trimer that has a typical mushroom shape; the TRAF-C domain forms the cap and TRAF-N coiled-coil domain forms the stalk (7, 31) (Figure 1E). Biochemical and structural analyses show that many interaction hot spots formed by β3, β4, β6, and β7 of the TRAF domains participate in the receptor interaction (Figure 1E). On the basis of the available structures of the trimeric TRAF domain, zinc-finger domain, and RING domain, a reconstituted full-length TRAF structure has been modeled (Figure 1F). Because there is no evidence of self-association between the zinc-finger domains or RING domains in the TRAF family, the C-terminal TRAF domain, which interacts with trimeric active receptors, forms a functional trimer, while the N-terminal RING domain and zinc-finger domain remain flexible (Figure 1F). In this regard, the length of the whole TRAF may be approximately 300Å and the shape is a long rod that is open at one end and closed at the opposite end.

## Receptor recognition by TRAFs

**Figure 2:**
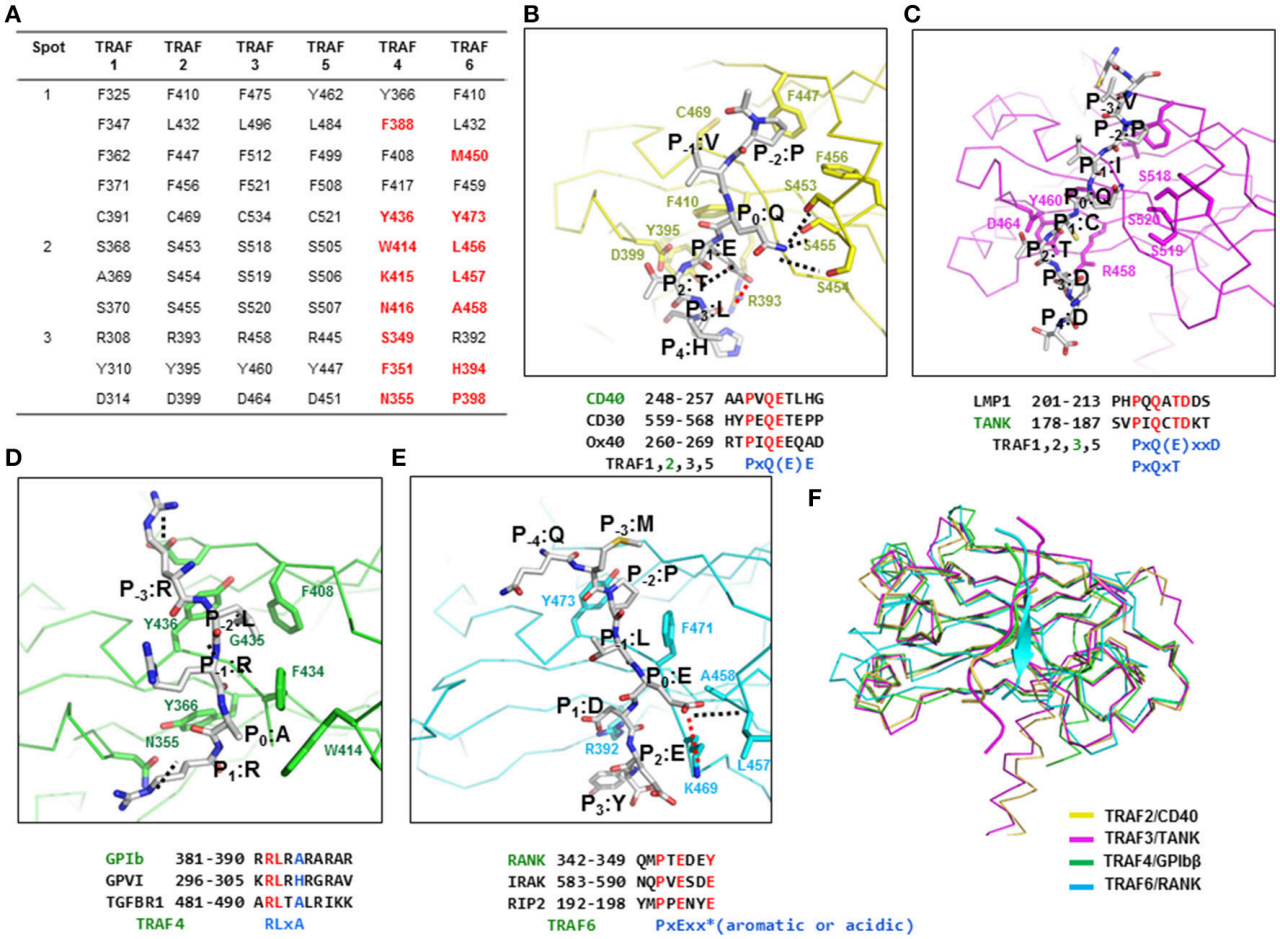
The TRAF-binding motif identified by structures of TRAF–receptor complexes. **(A)** Receptor-binding hot spots and conserved amino acid residues (in TRAF1, −2, −3, and −5) that are involved in the interaction with various receptors. The amino acid residues in TRAF4 and −6 that are not conserved are colored in red. **(B–E)** Detailed TRAF–receptor interaction. Close-up view of a CD40 peptide bound to TRAF2 **(B)**, a TANK peptide bound to TRAF3 **(C)**, a GPIbβ peptide bound to TRAF4 **(D)**, and a RANK peptide bound to TRAF6 **(E)**. Red dotted lines and black dotted lines indicate salt bridges and H-bonds, respectively. Amino acid positions of the peptide labeled as P_−4_-P_3_ are shown. TRAF-binding motifs are shown. **(F)** Structural comparison of the TRAF–receptor peptide complex by superposition analysis.

TRAF family members interact with various receptors and intracellular proteins, including CD40, CD30, Ox40, TRADD, LMP1, TNFR2, RANK, IRAK, RIP2, GPIb, GPVI, and TANK, during specific signaling events. The initial structural and biochemical studies on TRAFs (particularly TRAF2 and TRAF3) and their interacting receptors have revealed that for such interactions, TRAFs use three regions, known as binding hot spots: Hot spot 1, also known as the hydrophobic pocket, is composed of residues from β4, β5, β6, and β7. Hot spot 2, also known as the serine finger, is composed of three serine residues (one serine residue is replaced by alanine in TRAF1) from β6 and the loop connecting β6 and β7. Hot spot 3, also known as the polar pocket, is composed of polar residues from β3 and the loop connecting β3 and β4 (24, 25, 33, 34). The residues in these three hot spots of TRAF1, TRAF2, TRAF3, and TRAF5 are conserved, indicating that these TRAFs share receptor specificity via similar interaction modes (Figure 2A). The residues of TRAF4 and TRAF6 in the three binding hot spots of typical TRAFs are not conserved although several residues in hot spot 1 are conserved, indicating that TRAF4 and TRAF6 are unique and do not share binding mode and specificity to their receptors with typical TRAF family members (Figure 2A). Current available structures of TARF family with receptor peptides are listed at (Supplementary Table 3).

### TRAF1, TRAF2, TRAF3, and TRAF5

The typical binding hot spots and modes of interaction of TRAF family members have been well-studied for TRAF2 and TRAF3. The minimal consensus motif in TRAF-binding proteins, including TNF-R family members, CD30, CD40, Ox40, and LMP1, for TRAF2 and TRAF3 interaction is Px(Q or E)E# [x: any amino acid, #: acidic or polar amino acids are favored] (Figure 2B). Initial structural research on TRAF2 in complex with various peptides revealed the most conserved amino acid in the TRAF-binding motif to be P_0_, or the zero position of the TRAF-binding motif. Based on this labeling strategy, residues of the Px(Q or E)E motif were named as P (P_−2_), × (P_−1_), Q or E (P_0_), E (P_1_), and # (P2). For CD40, residues of the TRAF2-binding motif were named as P (P_−2_), V (P_−1_), Q (P_0_), E (P_1_), T (P_2_), and L (P_3_) (Figure 2B). To accommodate the Px(Q or E)E motif, hot spot 1–forming residues (F410, L432, F447, F456, and C469) in TRAF2 make extensive van der Waal contacts with P at the P_−2_ site. The major structural determinant of Q or E at the P_0_ position interacts with residues in hot spot 2 (serine triad, S453, S454, and S455 in TRAF2). Q at position P_0_ forms hydrogen bonds with all three serine residues, while E at the P_0_ position can form only one hydrogen bond. The carboxylate moiety of the Glu residue at position P_1_ engages in an ion-pair interaction with the side chain guanidinium group of R393 and forms a hydrogen bond with Y395 in TRAF2. All residues that are critical for the interaction with the Px(Q or E)E motif are completely conserved in TRAF1, TRAF3, and TRAF5, except that one serine residue in the serine triad is replaced by alanine in TRAF1 (A369), indicating that TRAF1, TRAF2, TRAF3, and TRAF5 share the same mode of interaction involving the Px(Q or E)E motif.

In addition to this major TRAF-binding motif [Px(Q or E)E motif], the minor motif [Px(Q/E)xxD motif] has been identified in several structural studies including the TRAF2–LMP1 (33) and TRAF3–TANK (35) complexes. Therefore, two minimal consensus motifs for binding to TRAF1, −2, −3, and −5, i.e., Px(Q/E)E as a major motif and Px(Q/E)xxD as a minor motif, have been identified. In the Px(Q/E)xxD motif, the side chains of the residues at positions P_−2_, P_0_, and P_3_ are critical for the TRAF interaction, unlike the major binding motif, where the side chains of residues at positions P_−2_, P_0_, and P_1_ participate in the interaction (Figure 2C). Several modified interactions involving the minor motif have been reported in structural studies on TRAF3 in complex with various receptors, including CD40 (26), LMP1 (36), and TANK (35) and the most recently solved TRAF1–TANK complex (37). In this case, the side chains of the P_−2_, P_0_, and P_2_ positions are involved in the TRAF interaction. The conserved amino acid residues at positions P_−2_, P_0_, and P_2_ are P at P_−2_, Q or E at P_0_, and T at the P_2_ position, forming the PxQxT consensus motif, which is considered an alternative minor TRAF1, −2, −3, and 5-binding motif. In the PxQxT motif, T (P_2_) interacts with the conserved aspartic acid residue (D314 in TRAF1 and D464 in TRAF3), whereas the interaction modes of P (P_−2_) and Q (P_0_) are similar to those of the major binding motif. Additionally, D in the minor consensus motif PxQxxD is not important for the interaction with TRAF. The recently solved TRAF1–TANK (SVPIQCTDKT) structure revealed sequence PxQxT as the TRAF1-binding motif (37). In this case, Q at the P_0_ position forms a hydrogen bond with S368 of TRAF1. C and T at positions P_1_ and P_2_, respectively, form hydrogen bonds with D314 of TRAF1.

### TRAF4

The TRAF4 structure was determined around the year 2013 by three research groups (28–30). On the basis of these structural studies, TRAF4 was identified as a lipid-binding protein that can modulate tight junctions involved in cell migration; abnormal overexpression of TRAF4 can induce carcinomas by affecting cell migration (29). In 2017, the TRAF4-binding motif was characterized in a study on a TRAF4–receptor complex (32). TRAF4 functions as an adaptor signaling molecule in platelet receptor–mediated production of reactive oxygen species by directly interacting with two platelet receptors: GPIb-IX-V and GPVI (38); peptides derived from these two platelet receptors have been used to analyze the TRAF4–receptor complex. The structure of the TRAF4–GPIbβ peptide complex has been solved by characterizing TRAF4 behavior and the TRAF4–platelet receptor interaction. If we use the same nomenclature, which denotes the most conserved amino acid position in the TRAF-binding motif as P_0_, or the zero position of the TRAF-binding motif, in the bound peptide from GPIbβ receptor, the residues in the RLRAR motif are named as R (P_−1_), L (P_0_), R (P_1_), A (P_2_), and R (P_3_) (Figure 2D). Although L (P_0_) is the most conserved amino acid position in the TRAF4-binding motif, more appropriate names for residues in the TRAF4-binding motif RLRAR are R (P_−3_), L (P_−2_), R (P_−1_), A (P_0_), and R (P_1_) according to previously identified receptor-binding hot spots (this notation is used hereafter). The first structure of a receptor complex of TRAF4 indicates that the side chains of R (P_−3_), L (P_−2_), A (P_0_), and R (P_1_) in GPIbβ receptor are involved in the TRAF4 interaction. The two hydrophobic pockets, major and minor, on the surface of TRAF4 are critical for its mode of receptor binding that is different from that of other TRAF family members. The major hydrophobic interaction is formed by L at P_−2_ of the GPIbβ peptide with F408, Y436, and F434 from TRAF4 and the second minor hydrophobic pocket is formed by A at P_0_ of GPIbβ with W414 and F434 from TRAF4 (Figure 2D). R at the P_−3_ position and another R at the P_1_ position form a hydrogen bond with the side chains of E406 and N355, respectively, from TRAF4. After further mutagenesis and interaction analyses using various peptides derived from putative TRAF4-binding receptors, the Arg-Leu motif at positions P_−3_ and P_−2_ was identified as crucial for the TRAF4 interaction, and the Ala residue at position P_0_ influences affinity. Replacement of Ala at position P_0_ with His (GPVI peptide) or Gly (TGF-β receptor 2 peptides) reduces the binding affinity for TRAF4, and replacement with Arg (NOD2 peptide) abrogates the interaction. This structural study estimated the TRAF4-binding motif for P_−3_ to P_0_ as Arg-Leu-X-Ala, where X can be any amino acid and Ala can be replaced by a small uncharged residue.

### TRAF6

The mode of interaction of TRAF6 with receptors is unique among TRAF family members and has been revealed by three available structures of complexes including TRAF6–CD40 (25), TRAF6–RANK (25), and TRAF6–MAVS (39). The TRAF6-binding motif is six amino acid residues in length, and the sequence is PxExxZ (x: any amino acid, Z: acidic or aromatic amino acid). Small hydrophobic resides can replace P. In accordance with the typical labeling system, the nomenclature of this motif is P (P_−2_), × (P_−1_), E (P_0_), × (P_−1_), × (P_−2_), and Z (P_−3_). The residues E235 of CD40, E346 of RANK, and E457 of MAVS have been designated as the P_0_ position of TRAF6-binding peptides. The receptor peptide residues corresponding to positions P_−4_ to P_3_ of CD40, RANK, and MAVS directly interact with TRAF6. Among the peptide residues, specific side chains of residues at P_−2_, P_0_, and P_3_ are the greatest contributors to these interactions. As observed in other TRAF family members, F471 and Y473 of TRAF6 form a hydrophobic pocket to accommodate the P residue at the P_−2_ position (Figure 2E). Possible replacement of P residue to A residue at P_−2_ position was studied by mutagenesis on CD40 (25). Because of the absence of the typical serine triad in TRAF6, E at position P_0_ in the receptor employs quite a different interaction strategy for incorporation into TRAF6. The carboxylate of E at the P_0_ position forms hydrogen bonds with the main chain amide nitrogen atoms of L457 and A458 and engages in an electrostatic interaction with the side chain of K469 (Figure 2E). This P_0_ interaction is also different from the TRAF4-binding motif in that TRAF4 uses a shallow hydrophobic pocket to bind to A at the P_0_ position of the receptor peptide. The residue at position P_3_ in CD40 (F238) and RANK (Y349) is adjacent to several aromatic and basic residues of TRAF6, including R392, forming an amino-aromatic interaction (Figure 2E).

## Concluding remarks

Despite the structural similarity of TRAF family members, each TRAF has specific biological functions with specificity to interacting partners: upstream receptors and downstream effector molecules. Because of the critical participation of the TRAF family in various signaling events, functional and structural analyses of these proteins have been conducted for several decades. Intensive studies have revealed that proteins of the TRAF family, except for TRAF7, contain a conserved TRAF domain at the C terminus, which mediates their interaction with upstream receptors and downstream effectors (3). Despite the structural similarity of the TRAF domain within the TRAF family, each domain of each TRAF protein is specific to interacting upstream receptors. In this review, we summarized the current understanding of TRAF-binding motifs of many receptors by examining the structures of all six TRAF family members and complexes of each TRAF with various receptors including recently characterized complexes TRAF4–GPIb (32), TRAF1–TANK (37), TRAF3–Cardif (27), and TRAF6–MAVS (39). Because the sequences of binding hot spots are conserved in TRAF1, −2, −3, and −5, they share the same binding consensus motifs, namely, one major motif, Px(Q/E)E, and two minor motifs: Px(Q/E)xxD, and Px(Q/E)xT. Although possessing nearly identical receptor binding motifs on TRAF2, −3, and −5, recent deep mutational scanning study with TRAF-peptide ligands showed key differences in binding preference. TRAF2, −3, and −5 have a binding preferences on CD40 and TANK with different affinity (40). The recently determined structure of TRAF4 in complex with its receptor GPIbβ revealed a novel mode of binding, which is consistent with the recently discovered receptor specificity of TRAF4, where nonconserved amino acid residues are critical for the interaction with various receptors. The TRAF4-binding motif is R (P_−3_), L (P_−2_), x, and A (P_0_) [RLxA motif], where x can be any amino acid and Ala can be replaced with a small uncharged residue. The TRAF domain of TRAF6 binds specifically to the consensus TRAF6-binding motif: PxExxZ (Z: an acidic or aromatic residue).

In conclusion, specificity of TRAFs can be mediated by different organization of binding hot spots. TRAF1, −2, −3, and −5, however, share various common receptors because of almost completely conserved binding hot spots. TRAF4 and TRAF6 are unique member of the TRAF family and have amino acid residues at the receptor-binding site that are completely different from those of other family members. As for the receptor interaction interface of TRAF4, the lower part of the receptor peptide (P_1_) binds to TRAF4 at a position similar to that of receptor peptides that bind to TRAF2 and TRAF3, whereas the upper part (P_−2_, and P_−1_) is far away from the receptor peptide–binding site in TRAF2 and TRAF3 (Figure 2F). Compared to the receptor-binding site of TRAF6, the receptor-binding sites of TRAF1, −2, −3, −4, and −5 do not overlap with the RANK peptide, which binds to TRAF6. Nonetheless, the P_−2_-binding pocket of TRAF6 is similar to that of other TRAFs, indicating that the region of receptor association in TRAF6 is slightly different from that of other TRAFs (Figure 2F). Because of the similarities and differences in the binding hot spots among TRAF family members, they can sometimes share receptors or select unique receptors in various important signaling pathways.

## Author contributions

The author confirms being the sole contributor of this work and approved it for publication.

### Conflict of interest statement

The author declares that the research was conducted in the absence of any commercial or financial relationships that could be construed as a potential conflict of interest.
