# Supplementary material for: Structure of TRAF Family: Current Understanding of Receptor Recognition
Source: Front Immunol. 2018 Aug 30;9:1999. doi: 10.3389/fimmu.2018.01999 (PMC6125299; doi:10.3389/fimmu.2018.01999)
Supplement: Supplementary file 1 [file Data_Sheet_1.docx]

Supplementary Material

**Structure of TRAF family: current understanding of receptor recognition**

Hyun Ho Park

^1^College of Pharmacy, Chung-Ang University, Seoul, Republic of Korea

Corresponding Author
xrayleox@cau.ac.kr

## Supplementary Figure1
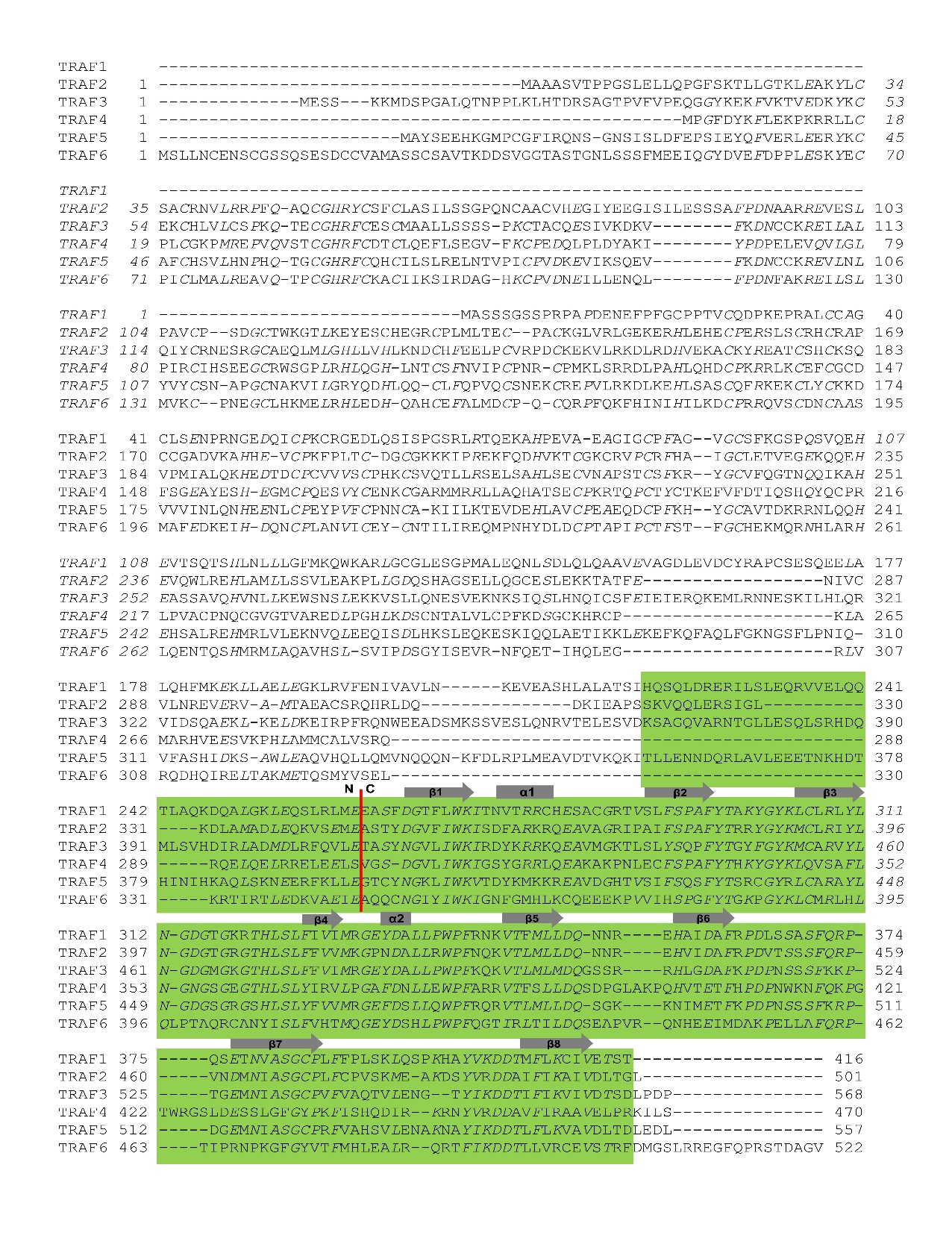


**Supplementary Figure 1.** Sequence alignment of TRAF family. TRAF domains are indicated by green high-right box. Red line indicates boundary between TRAF-N (N) and TRAF-C domain (C). Secondary structure was shown above the sequence.

## Supplementary Table1. Receptors with TRAF-binding motif

| TRAF family | TRAF1/2/3/5 | TRAF4 | TRAF6 |
| --- | --- | --- | --- |
| Receptors | TNFR2 | GPIb | CD40 |
|  | OX40 | GPVI | TACI |
|  | CD40 | TGFβR1 | RANK |
|  | CD30 | TGFβR2 | XEDAR |
|  | 4-1BB |  | TROY |
|  | RANK |  | BAFFR |
|  | HVEM |  | Fn14 |
|  | BCMA |  | IRAK |
|  | GITR |  | RIP2 |
|  | CD27 |  | OX40 |
|  | LMP1 |  | BCMA |
|  | RELT |  | TROY |
|  | LTbR |  | EDAR |
|  | TANK |  | p75 |
|  | TROY |  |  |
|  | EDAR |  |  |
|  | p75 |  |  |

## Supplementary Table2. Substrates of the E3 ligase activity of TRAFs

| TRAF family | Substrates | References |
| --- | --- | --- |
| TRAF2 | TRAF2 | (Habelhah et al., 2004) |
|  | TAK1 | (Fan et al., 2010) |
|  | RIP1 | (Alvarez et al., 2010) |
|  | TRIF | (Sasai et al., 2010) |
|  | cIAP1/2 | (Vallabhapurapu et al., 2008) |
|  | Smurf2 | (Carpentier et al., 2008) |
|  | Tristetraprolin | (Schichl et al., 2011) |
|  | DUSP14 | (Yang et al., 2016) |
|  | GβL | (Wang et al., 2017) |
| TRAF3 | TRAF3 | (Tseng et al., 2010) |
|  | TBK1 | (Netea et al., 2012) |
|  | ASC | (Guan et al., 2015) |
|  | IKK | (Netea et al., 2012) |
| TRAF4 | TRAF4 | (Wang et al., 2013) |
|  | Smurf1 | (Wang et al., 2013) |
|  | Smurf2 | (Zhang et al., 2013) |
|  | TAK1 | (Zhang et al., 2013) |
|  | TrkA | (Singh et al., 2018) |
|  | Akt | (Li et al., 2013) |
| TRAF5 | TRAF5 | (Tang and Wang, 2010) |
|  | RORγt | (Wang et al., 2015) |
| TRAF6 | TRAF6 | (Lamothe et al., 2008) |
|  | IL-17R | (Rong et al., 2007) |
|  | p75 | (Powell et al., 2009) |
|  | TGFβR1 | (Mu et al., 2011) |
|  | TAK1 | (Zhang et al., 2013) |
|  | IRAK1 | (Conze et al., 2008) |
|  | Akt | (Yang et al., 2009) |
|  | Fyn | (Liu et al., 2012) |
|  | NEMO | (Sebban-Benin et al., 2007) |
|  | NESCA | (Napolitano et al., 2009) |
|  | LAT | (Xie et al., 2013) |
|  | cIAP1/2 | (Tseng et al., 2010) |
|  | IRF5/7 | (Ning et al., 2008) |
|  | Beclin1 | (Shi and Kehrl, 2010) |
|  | NDP52 | (Inomata et al., 2012) |
|  | ECSIT | (West et al., 2011) |
|  | PIK3CA | (Wang et al., 2018) |
|  | PI3K (p85α) | (Hamidi et al., 2017) |

## Supplementary Table3. Current available structures of TRAF family with receptors

| TRAF family | Peptide receptor | PDB ID |
| --- | --- | --- |
| TRAF1(220-416) |  | 5E1T |
| TRAF1(220-416) | TANK(178-187) SVPIQCTDKT | 5H10 |
| TRAF2(327-501) |  | 1CA4 |
| TRAF2(327-501) | CD40(250-254) PVQET | 1D00 |
| TRAF2(327-501) | CD30(576-583) MLSVEEEG | 1D01 |
| TRAF2(327-501) | Ox40 (262-266) PIQEE | 1D0A |
| TRAF2(327-501) | m4-1BB (231-236) GAAQEE | 1D0J |
| TRAF2(327-501) | LMP1 (PQQATDD) | 1CZY |
| TRAF2(310-501) | CD40(250-254) PVQETLHGCQPVTQEDG | 1CZZ |
| TRAF2(310-501) | TNF-R2 (420-428) QVPFSKEEC | 1CA9 |
| TRAF3(341-568) |  | 1FLK |
| TRAF3(341-568) | CD40(247-268) TAAPVQETLHGCQPVTQEDG | 1FLL |
| TRAF3(377-568) | TANK(178-195) SVPIQCTDKTDKQEALFK | 1L0A |
| TRAF3(377-568) | TANK (171-191) IATDTQCSVPIQCTDKTDKQE | 1KZZ |
| TRAF3(377-568) | LTbR (385-408) PYPIPEEGDPGPPGLSTPHQEDGK | 1RF3 |
| TRAF3(377-568) | LMP1 (204-210) PQQATDD | 1ZMS |
| TRAF3(377-568) | BAFF-R (160-183) SVPVPATELGSTELVTTKTAGPEQ | 2GKW |
| TRAF3(377-568) | Cardif (138-158) PSCPKPVQDTQPPESPVENSE | 4GHU |
| TRAF4(290-470) TRAF4(292-466) |  | 4K8U, 4M4E |
| TRAF4(290-470) | GPIbβ (176-185) RRLRARARARA | 5YC1 |
| TRAF5(329-505) |  | 4GJH |
| TRAF6(346-504) |  | 1LB4 |
| TRAF6(346-504) | RANK (342-349) QMPTEDEY | 1LB5 |
| TRAF6(346-504) | CD40 (230-238) KQEPQEIDF | 1LB6 |
| TRAF6(346-504) | MAVS (450-468) GPCHGPEENEYKSEGTFGI | 4Z8M |

**References**

Alvarez, S.E., Harikumar, K.B., Hait, N.C., Allegood, J., Strub, G.M., Kim, E.Y., et al. (2010). Sphingosine-1-phosphate is a missing cofactor for the E3 ubiquitin ligase TRAF2. *Nature* 465(7301)**,** 1084-1088. doi: 10.1038/nature09128.

Carpentier, I., Coornaert, B., and Beyaert, R. (2008). Smurf2 is a TRAF2 binding protein that triggers TNF-R2 ubiquitination and TNF-R2-induced JNK activation. *Biochem Biophys Res Commun* 374(4)**,** 752-757. doi: 10.1016/j.bbrc.2008.07.103.

Conze, D.B., Wu, C.J., Thomas, J.A., Landstrom, A., and Ashwell, J.D. (2008). Lys63-linked polyubiquitination of IRAK-1 is required for interleukin-1 receptor- and toll-like receptor-mediated NF-kappaB activation. *Mol Cell Biol* 28(10)**,** 3538-3547. doi: MCB.02098-07 [pii]

10.1128/MCB.02098-07.

Fan, Y., Yu, Y., Shi, Y., Sun, W., Xie, M., Ge, N., et al. (2010). Lysine 63-linked polyubiquitination of TAK1 at lysine 158 is required for tumor necrosis factor alpha- and interleukin-1beta-induced IKK/NF-kappaB and JNK/AP-1 activation. *J Biol Chem* 285(8)**,** 5347-5360. doi: 10.1074/jbc.M109.076976.

Guan, K., Wei, C., Zheng, Z., Song, T., Wu, F., Zhang, Y., et al. (2015). MAVS Promotes Inflammasome Activation by Targeting ASC for K63-Linked Ubiquitination via the E3 Ligase TRAF3. *J Immunol* 194(10)**,** 4880-4890. doi: 10.4049/jimmunol.1402851.

Habelhah, H., Takahashi, S., Cho, S.G., Kadoya, T., Watanabe, T., and Ronai, Z. (2004). Ubiquitination and translocation of TRAF2 is required for activation of JNK but not of p38 or NF-kappaB. *EMBO J* 23(2)**,** 322-332. doi: 10.1038/sj.emboj.7600044.

Hamidi, A., Song, J., Thakur, N., Itoh, S., Marcusson, A., Bergh, A., et al. (2017). TGF-beta promotes PI3K-AKT signaling and prostate cancer cell migration through the TRAF6-mediated ubiquitylation of p85alpha. *Sci Signal* 10(486). doi: 10.1126/scisignal.aal4186.

Inomata, M., Niida, S., Shibata, K., and Into, T. (2012). Regulation of Toll-like receptor signaling by NDP52-mediated selective autophagy is normally inactivated by A20. *Cell Mol Life Sci* 69(6)**,** 963-979. doi: 10.1007/s00018-011-0819-y.

Lamothe, B., Campos, A.D., Webster, W.K., Gopinathan, A., Hur, L., and Darnay, B.G. (2008). The RING domain and first zinc finger of TRAF6 coordinate signaling by interleukin-1, lipopolysaccharide, and RANKL. *J Biol Chem* 283(36)**,** 24871-24880. doi: M802749200 [pii]

10.1074/jbc.M802749200.

Li, W., Peng, C., Lee, M.H., Lim, D., Zhu, F., Fu, Y., et al. (2013). TRAF4 is a critical molecule for Akt activation in lung cancer. *Cancer Res* 73(23)**,** 6938-6950. doi: 10.1158/0008-5472.CAN-13-0913.

Liu, A., Gong, P., Hyun, S.W., Wang, K.Z., Cates, E.A., Perkins, D., et al. (2012). TRAF6 protein couples Toll-like receptor 4 signaling to Src family kinase activation and opening of paracellular pathway in human lung microvascular endothelia. *J Biol Chem* 287(20)**,** 16132-16145. doi: 10.1074/jbc.M111.310102.

Mu, Y., Sundar, R., Thakur, N., Ekman, M., Gudey, S.K., Yakymovych, M., et al. (2011). TRAF6 ubiquitinates TGFbeta type I receptor to promote its cleavage and nuclear translocation in cancer. *Nat Commun* 2**,** 330. doi: 10.1038/ncomms1332.

Napolitano, G., Mirra, S., Monfregola, J., Lavorgna, A., Leonardi, A., and Ursini, M.V. (2009). NESCA: a new NEMO/IKKgamma and TRAF6 interacting protein. *J Cell Physiol* 220(2)**,** 410-417. doi: 10.1002/jcp.21782.

Netea, M.G., Wijmenga, C., and O'Neill, L.A. (2012). Genetic variation in Toll-like receptors and disease susceptibility. *Nat Immunol* 13(6)**,** 535-542. doi: 10.1038/ni.2284.

Ning, S., Campos, A.D., Darnay, B.G., Bentz, G.L., and Pagano, J.S. (2008). TRAF6 and the three C-terminal lysine sites on IRF7 are required for its ubiquitination-mediated activation by the tumor necrosis factor receptor family member latent membrane protein 1. *Mol Cell Biol* 28(20)**,** 6536-6546. doi: 10.1128/MCB.00785-08.

Powell, J.C., Twomey, C., Jain, R., and McCarthy, J.V. (2009). Association between Presenilin-1 and TRAF6 modulates regulated intramembrane proteolysis of the p75NTR neurotrophin receptor. *J Neurochem* 108(1)**,** 216-230. doi: 10.1111/j.1471-4159.2008.05763.x.

Rong, Z., Cheng, L., Ren, Y., Li, Z., Li, Y., Li, X., et al. (2007). Interleukin-17F signaling requires ubiquitination of interleukin-17 receptor via TRAF6. *Cell Signal* 19(7)**,** 1514-1520. doi: 10.1016/j.cellsig.2007.01.025.

Sasai, M., Tatematsu, M., Oshiumi, H., Funami, K., Matsumoto, M., Hatakeyama, S., et al. (2010). Direct binding of TRAF2 and TRAF6 to TICAM-1/TRIF adaptor participates in activation of the Toll-like receptor 3/4 pathway. *Mol Immunol* 47(6)**,** 1283-1291. doi: 10.1016/j.molimm.2009.12.002.

Schichl, Y.M., Resch, U., Lemberger, C.E., Stichlberger, D., and de Martin, R. (2011). Novel phosphorylation-dependent ubiquitination of tristetraprolin by mitogen-activated protein kinase/extracellular signal-regulated kinase kinase kinase 1 (MEKK1) and tumor necrosis factor receptor-associated factor 2 (TRAF2). *J Biol Chem* 286(44)**,** 38466-38477. doi: 10.1074/jbc.M111.254888.

Sebban-Benin, H., Pescatore, A., Fusco, F., Pascuale, V., Gautheron, J., Yamaoka, S., et al. (2007). Identification of TRAF6-dependent NEMO polyubiquitination sites through analysis of a new NEMO mutation causing incontinentia pigmenti. *Hum Mol Genet* 16(23)**,** 2805-2815. doi: 10.1093/hmg/ddm237.

Shi, C.S., and Kehrl, J.H. (2010). TRAF6 and A20 regulate lysine 63-linked ubiquitination of Beclin-1 to control TLR4-induced autophagy. *Sci Signal* 3(123)**,** ra42. doi: 10.1126/scisignal.2000751.

Singh, R., Karri, D., Shen, H., Shao, J., Dasgupta, S., Huang, S., et al. (2018). TRAF4-mediated ubiquitination of NGF receptor TrkA regulates prostate cancer metastasis. *J Clin Invest*. doi: 10.1172/JCI96060.

Tang, E.D., and Wang, C.Y. (2010). TRAF5 is a downstream target of MAVS in antiviral innate immune signaling. *PLoS One* 5(2)**,** e9172. doi: 10.1371/journal.pone.0009172.

Tseng, P.H., Matsuzawa, A., Zhang, W., Mino, T., Vignali, D.A., and Karin, M. (2010). Different modes of ubiquitination of the adaptor TRAF3 selectively activate the expression of type I interferons and proinflammatory cytokines. *Nat Immunol* 11(1)**,** 70-75. doi: 10.1038/ni.1819.

Vallabhapurapu, S., Matsuzawa, A., Zhang, W., Tseng, P.H., Keats, J.J., Wang, H., et al. (2008). Nonredundant and complementary functions of TRAF2 and TRAF3 in a ubiquitination cascade that activates NIK-dependent alternative NF-kappaB signaling. *Nat Immunol* 9(12)**,** 1364-1370. doi: ni.1678 [pii]

10.1038/ni.1678.

Wang, B., Jie, Z., Joo, D., Ordureau, A., Liu, P., Gan, W., et al. (2017). TRAF2 and OTUD7B govern a ubiquitin-dependent switch that regulates mTORC2 signalling. *Nature* 545(7654)**,** 365-369. doi: 10.1038/nature22344.

Wang, X., Jin, C., Tang, Y., Tang, L.Y., and Zhang, Y.E. (2013). Ubiquitination of tumor necrosis factor receptor-associated factor 4 (TRAF4) by Smad ubiquitination regulatory factor 1 (Smurf1) regulates motility of breast epithelial and cancer cells. *J Biol Chem* 288(30)**,** 21784-21792. doi: 10.1074/jbc.M113.472704.

Wang, X., Yang, J., Han, L., Zhao, K., Wu, Q., Bao, L., et al. (2015). TRAF5-mediated Lys-63-linked Polyubiquitination Plays an Essential Role in Positive Regulation of RORgammat in Promoting IL-17A Expression. *J Biol Chem* 290(48)**,** 29086-29094. doi: 10.1074/jbc.M115.664573.

Wang, Z., Liu, Y., Huang, S., and Fang, M. (2018). TRAF6 interacts with and ubiquitinates PIK3CA to enhance PI3K activation. *FEBS Lett* 592(11)**,** 1882-1892. doi: 10.1002/1873-3468.13080.

West, A.P., Brodsky, I.E., Rahner, C., Woo, D.K., Erdjument-Bromage, H., Tempst, P., et al. (2011). TLR signalling augments macrophage bactericidal activity through mitochondrial ROS. *Nature* 472(7344)**,** 476-480. doi: 10.1038/nature09973.

Xie, J.J., Liang, J.Q., Diao, L.H., Altman, A., and Li, Y. (2013). TNFR-associated factor 6 regulates TCR signaling via interaction with and modification of LAT adapter. *J Immunol* 190(8)**,** 4027-4036. doi: 10.4049/jimmunol.1202742.

Yang, C.Y., Chiu, L.L., and Tan, T.H. (2016). TRAF2-mediated Lys63-linked ubiquitination of DUSP14/MKP6 is essential for its phosphatase activity. *Cell Signal* 28(1)**,** 145-151. doi: 10.1016/j.cellsig.2015.10.017.

Yang, W.L., Wang, J., Chan, C.H., Lee, S.W., Campos, A.D., Lamothe, B., et al. (2009). The E3 ligase TRAF6 regulates Akt ubiquitination and activation. *Science* 325(5944)**,** 1134-1138. doi: 10.1126/science.1175065.

Zhang, L., Zhou, F., Garcia de Vinuesa, A., de Kruijf, E.M., Mesker, W.E., Hui, L., et al. (2013). TRAF4 promotes TGF-beta receptor signaling and drives breast cancer metastasis. *Mol Cell* 51(5)**,** 559-572. doi: 10.1016/j.molcel.2013.07.014.
